# Heart–gut interaction in veno-arterial extracorporeal membrane oxygenation: a forgotten axis in critical care perfusion

**DOI:** 10.1007/s10047-025-01543-6

**Published:** 2026-01-05

**Authors:** Amr Omar

**Affiliations:** Hamad Medical Corporation, Doha, Qatar

**Keywords:** GIT, Perfusion, VA-ECMO

## Abstract

The expanding use of veno-arterial extracorporeal membrane oxygenation (VA-ECMO) for cardiogenic shock and post-cardiotomy support necessitates a focus on perfusion beyond traditional targets. The gastrointestinal tract (GIT) is particularly vulnerable, yet its dysfunction is often overlooked in ECMO management. This editorial synthesizes current literature and pathophysiological principles to analyze the mechanisms of GIT injury during VA-ECMO support. We examine the impact of VA-ECMO on hemodynamics, microcirculation, and inflammation, with a focus on the heart-gut axis. VA-ECMO compromises gut integrity through multiple interconnected pathways. These include microcirculatory dysfunction due to non-pulsatile flow and vasopressor use, increased left ventricular afterload, and ischemia-reperfusion injury. The phenomenon of “dual circulation” in peripheral VA-ECMO creates a risk of heterogeneous oxygen delivery, potentially leaving the GIT hypoperfused. These insults lead to mucosal barrier failure, bacterial translocation, and systemic inflammation, driving sequential organ failure. Early recognition of gut dysfunction, through clinical monitoring and biomarkers, is challenging but critical. Given the high morbidity and mortality associated with VA-ECMO, the GIT must be acknowledged as a critical target organ. A paradigm shift integrating gut-protective strategies, such as meticulous fluid management, enteral nutrition, and monitoring for signs of hypoperfusion into standard VA-ECMO protocols is essential to improve patient outcomes.

## Introduction

The growing use of venoarterial extracorporeal membrane oxygenation (VA-ECMO) in cardiogenic shock (CS) and post-cardiotomy support has renewed interest in the perfusion dynamics of organs beyond the heart and brain. Among these, the gastrointestinal tract (GIT) has emerged as a vulnerable and under-recognized contributor to sequential organ failure. In patients supported with VA-ECMO, heart–gut interactions arise through intertwined hemodynamic, microcirculatory, and inflammatory mechanisms.

Peripheral VA-ECMO typically returns oxygenated blood retrograde through the femoral artery, elevating systemic arterial pressure and overall cardiac output, but at the expense of increased left ventricular afterload. These complex flow dynamics can influence regional organ perfusion, including splanchnic circulation, and may contribute to downstream GIT injury [1, 2]. Gastrointestinal tract disorders, particularly UGIB, are a significant complication in patients receiving VA-ECMO. The incidence of UGIB during VA-ECMO is consistently reported at approximately 10% in large cohort studies, with median onset around 10–12 days after cannulation [1, 2]. UGIB is associated with substantial morbidity, including increased requirements for blood product transfusion, prolonged duration of mechanical ventilation and ECMO support, and extended intensive care unit and hospital stays [3, 4]. Mortality rates among VA-ECMO patients who develop UGIB are notably high. In one cohort, 75% of patients with UGIB died during hospitalization, compared to 60% mortality in those without UGIB [4]. 

## Materials and methods

This report is based on a narrative literature review, synthesizing:

- Experimental and clinical studies on GIT perfusion in VA-ECMO.
- Reports on splanchnic microcirculation, ischemia–reperfusion injury, and intestinal barrier dysfunction.
- Observational studies on gastrointestinal bleeding, mucosal injury, and inflammatory biomarkers in VA-ECMO patients.
- Foundational physiological descriptions of flow competition (“dual circulation”) in peripheral VA-ECMO circuits.

Search terms included VA-ECMO, splanchnic circulation, gastrointestinal injury, microcirculation, ischemia–reperfusion, and dual circulation.

No human subjects, interventions, or patient-level data were collected.

## Results

1. Hemodynamic and Microcirculatory Changes

Although VA-ECMO increases mean arterial pressure and total cardiac output, multiple studies demonstrate:

- Reduced hepatic and intestinal microcirculatory oxygen saturation
- Decreased microvascular hemoglobin concentration
- Despite normalized systemic hemodynamics, splanchnic perfusion is often impaired [3–5]

Contributing factors:

- Loss of pulsatile flow
- Vasopressor-induced vasoconstriction
- Venous congestion
- Flow redistribution away from the splanchnic bed
- Inflammatory activation from extracorporeal circulation [5–7]

2. Ischemia–Reperfusion Injury and GIT Bleeding

GIT bleeding is a recognized complication of VA-ECMO. Established risk factors include:

- Prior peptic ulcer disease
- Extracorporeal cardiopulmonary resuscitation (ECPR)
- Dual antiplatelet therapy

Microcirculatory impairment worsens mucosal ischemia–reperfusion injury, leading to inflammatory activation and systemic biomarker release [5–7].

3. Flow Competition (“Dual Circulation”)

Bachmann et al. described the phenomenon of dual circulation, in which:

- ECMO produces retrograde aortic flow
- Native heart produces antegrade aortic flow
- The “mixing zone” determines which organs receive oxygen-rich ECMO blood

Regional oxygenation to abdominal organs may vary widely depending on relative ECMO versus native cardiac output [2]

4. Additional Factors Contributing to GIT Injury

- Non-pulsatile flow and vasopressor dependence impair splanchnic perfusion [4, 8]
- Delayed enteral nutrition exacerbates mucosal injury and worsens outcomes [9]
- Gut barrier disruption allows bacterial translocation, fueling systemic inflammation [5, 6, 10]

5. Clinical Recognition

Indicators of impaired GIT perfusion include:

- Rising lactate
- Abdominal distension
- Altered bowel sounds
- High gastric residuals
- Stool changes
- Biomarkers of barrier disruption and inflammation

6. Biomarkers

Early detection of gastrointestinal injury during VA-ECMO is essential, and biomarkers may play a key role in both diagnosis and prognosis.

Alterations in cytokine profiles have been consistently linked to intestinal injury in patients receiving VA-ECMO, as demonstrated in both experimental and clinical studies. VA-ECMO triggers a pronounced and rapid systemic inflammatory response, reflected by elevations in pro-inflammatory mediators such as IL-6, IL-8, and TNF-α, along with increases in the anti-inflammatory cytokine IL-10 [11, 12]. 

Findings from a rat model of septic shock show that, despite improvements in systemic blood pressure and cardiac output, VA-ECMO does not necessarily restore intestinal microcirculatory flow. Instead, microvascular disturbances may worsen intestinal hypoperfusion and tissue injury. Chemokines such as CXCL2 appear to vary with ECMO flow rates—higher flows increase CXCL2 expression, while lower flows are associated with heightened pulmonary inflammation—highlighting a complex relationship between systemic and organ-specific inflammatory activity [5]. In addition, increased intestinal permeability and a heightened risk of downstream organ dysfunction have been attributed to endothelial activation and injury. VA-ECMO has been associated with upregulation of endothelial markers (e.g., ICAM-1, selectins) and elevated circulating indicators of endothelial damage (e.g., angiopoietin-2, von Willebrand factor). These changes promote vascular leakage, edema, and further compromise of gut barrier function [13]. 

Intestinal fatty acid–binding protein (I-FABP) is a well-recognized marker of enterocyte damage and gastrointestinal dysfunction. Although the ECMO literature does not directly examine I-FABP concentrations during extracorporeal support, evidence from cardiac surgery patients undergoing cardiopulmonary bypass—a setting that involves similar ischemia-reperfusion and inflammatory mechanisms—shows a consistent postoperative increase in I-FABP levels [14]. – [15] Higher I-FABP concentrations have been linked to more severe gastrointestinal injury and are associated with greater risks of organ dysfunction and mortality [15]. 

## Discussion

The gastrointestinal tract is particularly susceptible to injury during VA-ECMO due to the mismatch between global hemodynamic improvement and regional microcirculatory impairment. These changes compromise intestinal barrier integrity, promoting bacterial translocation and fueling systemic inflammation, which can worsen multiorgan dysfunction.

Despite these risks, the GIT is often overlooked in VA-ECMO management. Standard protocols typically emphasize cardiac output, MAP, and oxygen delivery without integrating splanchnic microcirculatory assessment.

Gastrointestinal dysfunction is increasingly recognized as a significant contributor to morbidity during VA-ECMO. Although VA-ECMO improves global hemodynamics, accumulating evidence shows that its effects on regional perfusion—particularly within the gastrointestinal tract—are complex and often unfavorable.

Recent experimental data demonstrate that VA-ECMO can impair hepatic and intestinal microcirculation even when systemic blood flow and blood pressure are optimized. Reduced microvascular oxygen saturation and hemoglobin concentration have been observed independent of ECMO flow rates, with dilutional anemia and systemic inflammation contributing to these abnormalities. These findings suggest that VA-ECMO may worsen splanchnic hypoperfusion rather than consistently improving it, particularly in the setting of septic shock [3]. 

Clinical studies further highlight the gastrointestinal tract as a vulnerable organ during VA-ECMO. Upper gastrointestinal bleeding (UGIB) occurs in approximately 10% of adults supported with VA-ECMO, most commonly from gastroduodenal ulcers. UGIB is associated with prolonged ICU and hospital length of stay and increased mortality. Identified risk factors include a history of peptic ulcer disease, dual antiplatelet therapy, and the use of extracorporeal cardiopulmonary resuscitation. The combination of ischemia–reperfusion injury, anticoagulation, and antiplatelet therapy likely contributes to mucosal vulnerability [5]. 

Nutritional challenges further underscore the burden of gastrointestinal dysfunction in VA-ECMO patients. Observational data show that nutrition adequacy is significantly reduced in the early days of VA-ECMO support compared with VV-ECMO. Positive fluid balance, need for vasopressors/inotropes, and elevated bilirubin levels appear to impede effective nutritional delivery, suggesting early hepatic and gastrointestinal impairment is common [16]. 

Volume management also plays a critical role. Animal studies indicate that excessive fluid administration increases intestinal edema, which can compromise mucosal integrity and exacerbate gut-related complications. Notably, this edema appears more pronounced in the bowel than in other organs, highlighting the sensitivity of the gastrointestinal tract to even modest fluid shifts during VA-ECMO [17]. 

Finally, a recent meta-analysis confirms that VA-ECMO carries a higher risk of major bleeding complications—including gastrointestinal bleeding—as well as ischemic vascular events. These findings reinforce the need to balance anticoagulation strategies with bleeding risk and to incorporate early screening and preventive measures for gastrointestinal complications [18]. 

### Protective measures

Intestinal protection during VA-ECMO management is a critical concern.

First, routine prophylactic measures such as proton pump inhibitor (PPI) therapy and early enteral nutrition are commonly employed, but recent evidence suggests these interventions may not be sufficient to prevent UGIB in VA-ECMO patients. Despite high rates of PPI use and enteral feeding, the incidence of UGIB remains approximately 10%, and neither intervention has demonstrated clear protective benefit in this setting [3]. 

Second, risk stratification is essential. Patients with a history of peptic ulcer disease, those receiving dual antiplatelet therapy, and those undergoing ECPR are at significantly increased risk for GI bleeding [4]. Identification of these risk factors should prompt heightened surveillance and consideration of more aggressive prophylactic or diagnostic strategies.

Third, early recognition and prompt investigation of suspected GI injury are crucial. The literature supports a low threshold for performing esophagogastroduodenoscopy (EGD) in VA-ECMO patients with clinical suspicion of GI bleeding, as overt signs may be masked by sedation, mechanical ventilation, and the effects of extracorporeal support [3, 4]. Regular monitoring of hemoglobin, hematocrit, and occult blood in gastric aspirates or stool can aid in early detection.

Fourth, multidisciplinary management—including close collaboration between intensivists, gastroenterologists, and surgeons—is recommended for timely diagnosis and intervention. Endoscopic therapy is often required for ulcerative lesions, which are the most common source of bleeding in this population [4]. 

Finally, ongoing research is needed to clarify the optimal approach to GI protection in VA-ECMO patients, as current prophylactic strategies may be inadequate. Until more definitive evidence is available, individualized risk assessment, vigilant monitoring, and early diagnostic intervention remain the cornerstones of intestinal protection.

### Future priorities should include

- Incorporating gut perfusion markers into routine VA-ECMO monitoring
- Earlier initiation of enteral nutrition when safe
- Reducing unnecessary vasopressor exposure
- Optimizing ECMO flow and pulsatility
- Utilizing ultrasound or NIRS for bedside splanchnic assessment

## Conclusion

The gastrointestinal tract is a critical yet frequently under-recognized organ in patients supported with VA-ECMO. Despite improvements in systemic hemodynamics, splanchnic microcirculation may remain severely compromised, predisposing patients to mucosal injury, bleeding, bacterial translocation, and inflammatory amplification.

Integrating gut-directed monitoring and protective strategies into VA-ECMO management protocols is essential. These efforts require coordinated involvement from intensivists, surgeons, perfusionists, and nutrition specialists to mitigate gastrointestinal complications and improve overall survival.

## Data Availability

Data sharing is not applicable to this article as it is a brief communication based on expert opinion and review of the published literature, and no new data were generated or analyzed in this study.

## Abbreviations

CS: Cardiogenic shock
ECPR: Extracorporeal cardiopulmonary resuscitation
GIT: Gastrointestinal tract
UGIB: Upper gastrointestinal bleeding
VA-ECMO: Veno:arterial extracorporeal membrane oxygenation
